# Correction: Composition of nasal bacterial community and its seasonal variation in health care workers stationed in a clinical research laboratory

**DOI:** 10.1371/journal.pone.0292828

**Published:** 2023-10-09

**Authors:** Nazima Habibi, Abu Salim Mustafa, Mohd Wasif Khan

There are errors in the Funding section. The correct Funding statement is: The study was funded by Kuwait University Research Sector’s grants RM01/13 and SRUL02/13.
