# Supplementary material for: Composition of nasal bacterial community and its seasonal variation in health care workers stationed in a clinical research laboratory
Source: PLoS One. 2021 Nov 24;16(11):e0260314. doi: 10.1371/journal.pone.0260314 (PMC8612574; doi:10.1371/journal.pone.0260314)
Supplement: S1 Appendix — (PDF) [file pone.0260314.s001.pdf]

Supplementary Data -Nasal Microbiota and its Seasonal Variation in Health CareWorkers Stationed in a Clinical Research Laboratory

Quality assessment during library preparation

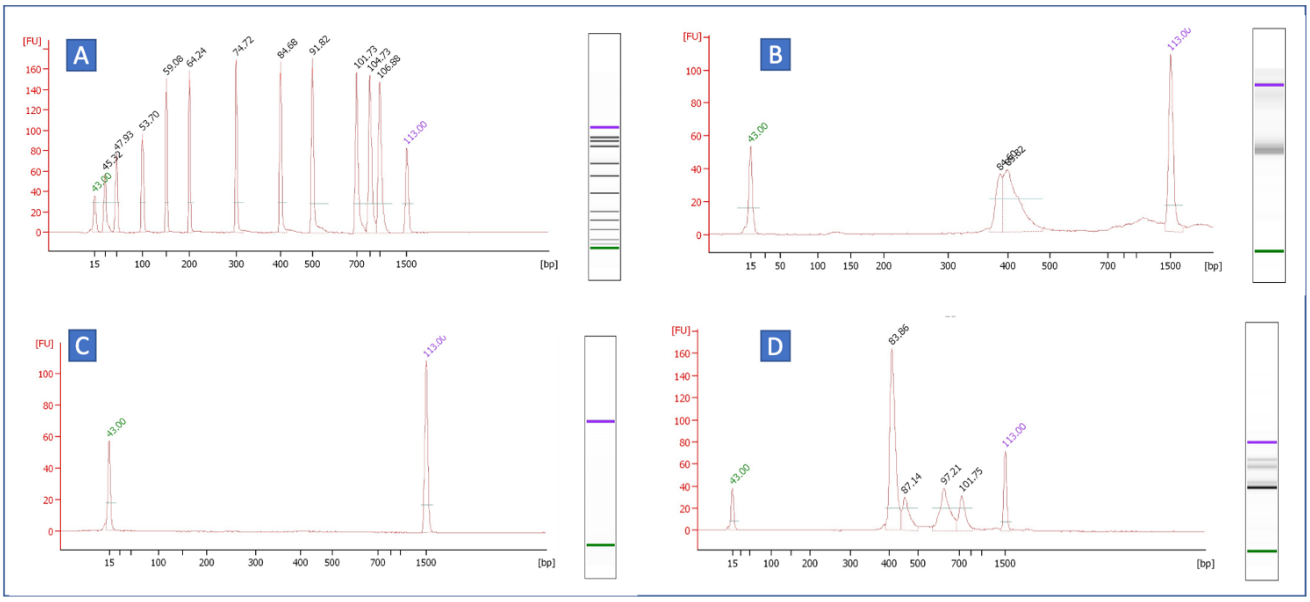

Fig S1. Bioanalyzer traces of (a) DNA 1000 marker; (b) Positive control *E. coli* DNA; (c) Negative control -nuclease free water; (d) representative sample library.

Table S1. Basic Statistics and quality scores of sequences submitted at MG-Rast server

S. No.	MG-RAST ID	name	bp count	Sequence count	post QC bp count	low quality	predicted feature	*Phred Score	*%GC
1	146348c2556d676d343638313233342e33	07_Nov_1105	85066775	339129	2,765,837	20,921	357,169	31	54
2	21aa50a9e46d676d343638313232382e33	13_Nov_1108	70453722	280374	2,375,899	20,348	275,393	30	52
3	2303ad67996d676d343638313232392e33	08_Nov_1107	63663728	253652	1,823,011	13,895	243,115	34	53
4	4c6339b3c96d676d343638313233352e33	16_Nov_1114	5859175	23448	575,444	1,842	24,094	30	53
5	59de68aa156d676d343638313233312e33	04_Nov_1110	120115310	482008	4,221,723	37,848	470,211	35	55
6	5b6ed648376d676d343638313233322e33	15_Nov_1113	430861464	1717978	9,092,782	131,657	1,652,226	30	52
7	60feec6efe6d676d343638313233382e33	02_Nov_1103	82535544	328608	3,168,054	19,751	344,137	34	54
8	62df607e456d676d343638313234302e33	06_Nov_1109	46811064	186743	1,756,452	13,103	178,114	28	51
9	83ff00be1d6d676d343638313233302e33	05_Nov_1101	27104031	107926	1,528,707	9344	107115	35	52
10	8a33bf0d1a6d676d343638313233392e33	09_Nov_1106	59733057	237911	1,900,117	14,159	233,033	30	51
11	a685f61e6d6d676d343638313233332e33	03_Nov_1102	87665617	348721	3,296,371	21,903	362,760	35	53
12	b1892451c96d676d343638313233362e33	14_Nov_1111	153106102	612259	4,221,723	37848	470,211	31	51
13	c0fed74d856d676d343638313233372e33	01_Nov_1104	30289624	121097	1,649,014	10,857	130,423	35	52
14	fa743c96836d676d343638313232372e33	10_Nov_1112	32360797	129773	1,554,258	12,284	141,287	31	54
1	0ee0718b546d676d343638313336392e33	08_Dec_1207	83568037	333582	2,209,582	21,870	364,128	35	52
2	2507b815b96d676d343638313337392e33	03_Dec_1205	113065036	449985	2,502,671	26,160	469,972	31	53
3	2d813525276d676d343638313338322e33	09_Dec_1206	45312494	181408	1,782,079	16,887	183,524	34	51
4	38cc99fff26d676d343638313336382e33	02_Dec_1201	67744079	270703	1,788,146	16,397	286,006	30	55
5	50b3588b426d676d343638313336362e33	14_Dec_1213	230112286	916358	5,238,442	48,041	924,437	34	54
6	635ba409ef6d676d343638313337362e33	01_Dec_1218	63767407	254039	1,835,607	15,748	269,318	30	54
7	734967ec226d676d343638313336372e33	15_Dec_1214	34439121	137951	1,492,554	10,697	142,956	34	51
8	787f91d62e6d676d343638313337352e33	18_Dec_1217	60233603	240157	2,539,696	57,136	177,121	30	54
9	7b9cd1ccd36d676d343638313337322e33	10_Dec_1210	72641922	290179	2,020,950	23,159	336,172	35	51
10	95f38e24226d676d343638313337312e33	17_Dec_1216	197562442	787327	4,121,379	48,188	961,127	30	54
11	be7eb8e4c96d676d343638313338312e33	04_Dec_1203	39995258	159575	1,407,106	10,920	169,315	35	51
12	c0883e38846d676d343638313337372e33	05_Dec_1208	90559029	360754	2,427,075	23,106	382,596	30	55
13	d02af31e566d676d343638313337302e33	12_Dec_1204	89913430	358380	2,542,726	22,986	383,415	35	51
14	df624c968e6d676d343638313337342e33	11_Dec_1211	12016040	47892	679,023	5,774	48,879	28	54
15	eb599e1ca76d676d343638313337382e33	06_Dec_1209	96751522	385781	2,597,145	23,224	405,962	35	51
16	f052693c3a6d676d343638313338302e33	13_Dec_1212	99310723	396173	2,679,093	23,949	418,267	30	55
17	f11467951c6d676d343638313337332e33	07_Dec_1202	61416127	244700	1,731,438	15,745	276,500	35	51
1	0cad72f9016d676d343638313636332e33	07_Feb_0208	50228377	200553	1,394,571	13,247	268,944	28	54
2	0d6f55bb216d676d343638313635372e33	10_Feb_0205	138097566	551833	2,502,561	43,661	541,840	35	51
3	0d894a364f6d676d343638313635332e33	12_Feb_0207	74015035	295151	1,850,737	20,956	274,517	28	55
4	110025b7cc6d676d343638313634372e33	04_Feb_0218	146714854	588584	5,073,851	49,850	726,130	35	51
5	1290c773e96d676d343638313635392e33	08_Feb_0219	65770890	262541	1,746,171	16,983	410,337	30	54
6	3536c6352f6d676d343638313635342e33	15_Feb_0209	27119200	108071	1,394,447	9,223	125,553	35	51
7	53079188ba6d676d343638313636312e33	20_Feb_0217	35515636	141376	1,162,408	10,038	130,502	30	55
8	64d7acd4666d676d343638313635352e33	22_Feb_0212	50500952	201688	1,717,534	15,571	259,123	35	55
9	69d22cf3a56d676d343638313635362e33	17_Feb_0213	73219480	292403	2,064,929	19,167	395,850	30	54
10	742243ce866d676d343638313636342e33	19_Feb_0216	53791453	214892	1,247,183	17,455	255,643	34	52
11	75163f975e6d676d343638313636382e33	02_Feb_0204	54682624	218293	1,600,851	16,123	452,687	30	55
12	75f839f8776d676d343638313636372e33	14_Feb_0206	37948550	154087	1,908,110	12,300	175,727	33	51
13	7c083b4da46d676d343638313635312e33	05_Feb_0201	37551333	149535	1,457,577	9,297	142,733	30	53
14	9115d322696d676d343638313636302e33	09_Feb_0211	159676292	636183	3,515,715	35,650	626,265	35	51
15	92beb441076d676d343638313636352e33	01_Feb_0214	82397253	328895	2,322,532	22,415	363,125	30	51
16	ab156b93826d676d343638313634382e33	18_Feb_0215	83345451	333988	1,947,265	21,450	377,988	35	55
17	ba9e58cab46d676d343638313636362e33	21_Feb_0221	50348125	200577	1,223,899	11,701	192,279	28	52
18	bc034fbcd46d676d343638313635322e33	03_Feb_0222	124763614	497229	2,831,336	33,536	487,803	35	56
19	daf8b3605e6d676d343638313634392e33	16_Feb_0210	121607532	485133	2,474,675	30,787	817,573	30	53
20	e3ac9657d66d676d343638313635302e33	06_Feb_0203	116517458	463949	2,227,447	24,128	582,451	35	52
21	e3c8d6a5806d676d343638313635382e33	11_Feb_0220	18797183	79118	1,301,465	10,878	92,891	29	51
22	f1a4c183ee6d676d343638313636322e33	13_Feb_0202	90624821	361588	2,092,626	23,987	458,756	30	54
1	2356e4a32e6d676d343638323434332e33	06_Apr_2014	102298051	307703	4,106,322	71,819	229,022	30	51
2	3db6913c6e6d676d343638323434342e33	04_Apr_2014	150492395	411588	3,437,396	84,326	362,950	35	52
3	582820f92a6d676d343638323434352e33	02_Apr_2014	75054605	219437	2,858,795	54,457	187,221	31	56
4	792dc7543b6d676d343638323434382e33	03_Apr_2014	31695527	95161	1,468,089	26,199	56,138	28	53
5	a1e08aff5b6d676d343638323434362e33	01_Apr_2014	51116254	144219	1,451,679	48,124	96,742	24	52
6	bf400509546d676d343638323434372e33	05_Apr_2014	137707951	392594	3,863,533	86,514	314,009	26	51
1	1a47e26d816d676d343638323433392e33	05_Jun_2014	56372233	224066	2,321,705	14,922	271,498	34	52
2	253b85a6026d676d343638323433362e33	02_Jun_2014	136634244	402924	4,035,045	100,349	382,719	30	50
3	2ee2afc58a6d676d343638323433312e33	01_Jun_2014	39084094	117875	1,949,430	30,086	100,661	32	53
4	35487e94506d676d343638323433382e33	09_Jun_2014	146929802	419918	3,914,513	103,221	347,182	34	54
5	476efca31c6d676d343638323434312e33	15_Jun_2014	77627137	233350	3,054,384	65,145	150,654	30	54
6	7be7945d196d676d343638323433322e33	11_Jun_2014	134681715	388639	4,061,133	82,153	375,085	34	52
7	7d62d0e2e16d676d343638323434302e33	07_Jun_2014	32116372	127896	1,747,589	8,878	132,662	30	54
8	861b278bb06d676d343638323433302e33	17_Jun_2014	82584274	249663	3,191,599	68,068	216,223	34	53
9	92be3cbd786d676d343638323432392e33	04_Jun_2014	92053718	265613	2,646,782	55,697	262,371	30	54
10	a1b020c5006d676d343638323433342e33	08_Jun_2014	45589346	181563	1,744,373	12,955	191,410	36	56
11	a3b0d8b6996d676d343638323433332e33	16_Jun_2014	13195932	38451	773,904	12,757	26,209	35	52
12	d466ba6d0f6d676d343638323434322e33	10_Jun_2014	63654287	183637	1,746,160	46,272	143,910	33	51
13	db2a1d95466d676d343638323433372e33	06_Jun_2014	51520220	152521	2,017,793	42,662	128,113	36	53
14	e7e362cb6e6d676d343638323433352e33	13_Jun_2014	61777166	180517	2,133,782	45,818	195,764	35	50

The Phred score and the % GC were obtained through the Fastqc. (Andrews et al.,)

Library size check

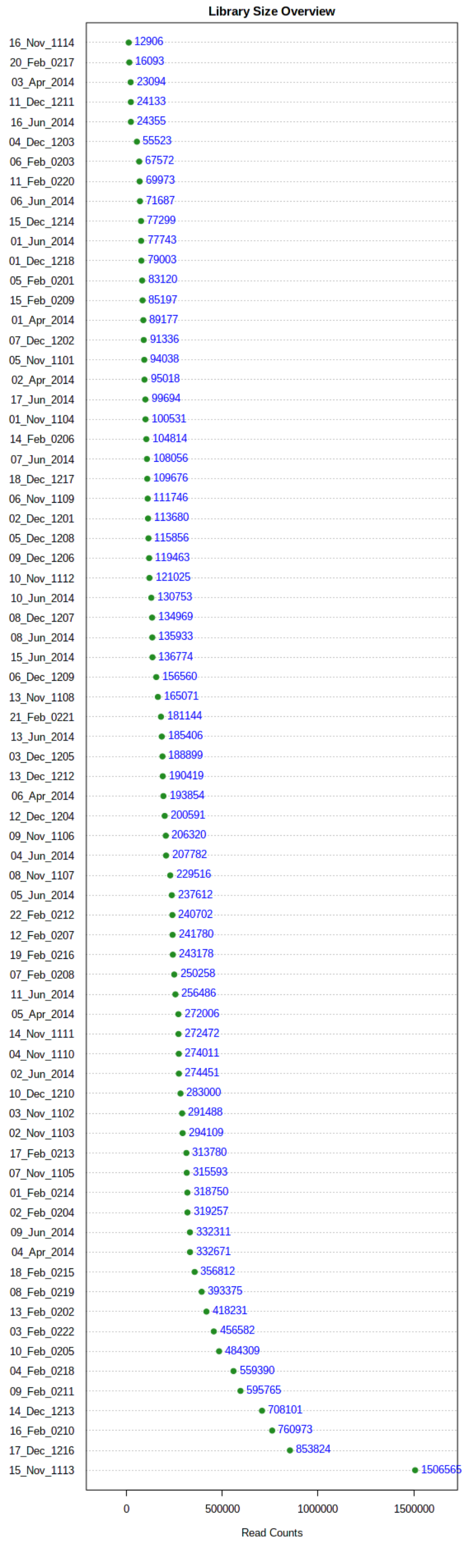

Fig S2. Raw read counts of n=73 samples after adapter trimming and removal of duplicate reads.

Rarefaction Analysis

A look at the library size overview suggested more than 10X size difference between the datasets of 73 samples. Therefore, rarefaction analysis was performed. The data were rarified at 11733 bp. A plateau was observed >5000 bp. The Good's coverage averaged at 99.85% for all the libraries.

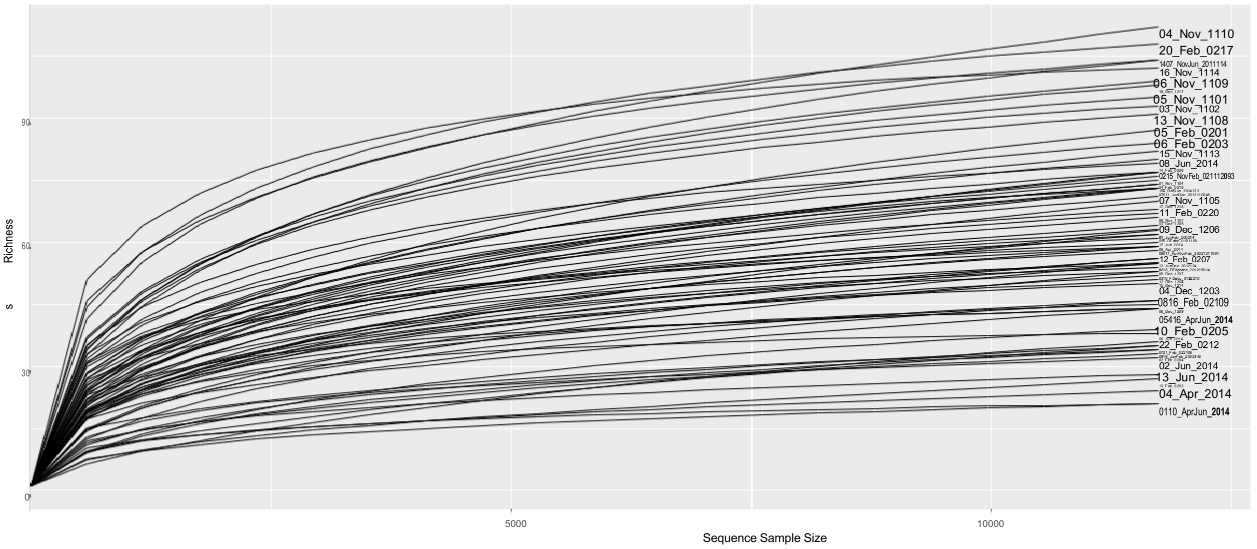

Fig S3. Rarefaction curves and alpha diversity scores based on normalized sequence abundance counts depicting overall species richness of all metagenomes. The x-axis represents the number of sequence reads while the y-axis represents the species counts.

Comparative Analysis with two publicly available datasets

We compared our dataset with two control groups. The first group comprised of 88 professionals working in health care institutes in China (HCC). The second group of 20 healthy individuals (not associated with animals) working in a live-stock farm in Iowa (HC). Both the studies conducted 16s amplicon sequencing and generated 1683 and 262 OTUs respectively. A combined OTU file including 2363 OTUs from our dataset was created. All the OTUs were converted to RA and normalized through cumulative sum scaling (CSS). We performed the taxonomic profiling uptogenus level. We found differences at genus level. Predominant genera identified in all the studies were *Corynebacterium*, *Staphylococcus, Moraxella* with varied relative abundances. Genera such as *Agrobacterium, Bacillus, Cytophaga, Flavobacterium, Lactobacillus, Myroides, Pseudomonas, Shigella, Sinorhizobium* were not found in the NHC group. The Linear Discriminant Analysis (LDA) revealed 86 genera to be significantly differentially abundant (FDR-q < 0.05) between the three studies. LDA scores of top twenty genera are listed in the table below.

Table S2. Comparative Analysis of our dataset with two publicly available datasets (HCC- professionals working in health care institutes in Taiwan (Chen et al., 2019) and NHC- healthy individuals working in a livestock farm in Iowa (Kates et al., 2019)

	HC RA %	HCC RA %	NHC RA %	P-values	FDR	LDA Score
Acinetobacter	0.018	0.000	0.001	3.2546E-29	8.1366E-28	3.94
Agrobacterium	0.005	0.000	0.000	2.7315E-25	4.2273E-24	3.39
Bacillus	0.005	0.000	0.000	5.4444E-38	7.0777E-36	3.39
Corynebacterium	0.137	0.021	0.070	8.6011E-33	4.6589E-31	4.82
Cytophaga	0.010	0.000	0.000	1.1634E-31	4.2011E-30	3.7
Dolosigranulum	0.000	0.000	0.150	4.5827E-16	3.203E-15	3.8
Flavobacterium	0.008	0.000	0.000	2.4622E-32	1.0669E-30	3.61
Lactobacillus	0.013	0.000	0.000	1.2566E-33	7.4252E-32	3.79
Moraxella	0.147	0.027	0.093	2.3223E-22	2.7381E-21	4.85
Myroides	0.006	0.000	0.000	5.4092E-31	1.758E-29	3.48
Peptoniphilus	0.006	0.000	0.016	1.8643E-26	3.366E-25	3.47
Propionibacterium	0.003	0.000	0.214	3.4281E-37	3.1832E-35	3.96
Pseudomonas	0.026	0.085	0.000	7.8801E-21	8.0032E-20	4.11
Shigella	0.011	0.000	0.000	6.0434E-18	4.6214E-17	3.72
Sinorhizobium	0.005	0.000	0.000	4.2908E-24	5.6918E-23	3.38
Staphylococcus	0.065	0.051	0.269	1.639E-28	3.4367E-27	4.5
**Others**	0.055	0.815	0.185			

The data set was normalized through centered log ratio (CLR) algorithm to perform the betadiversity clustering. The outcomes were plotted on a PCoA plot. The PCoA analysis on threedatasets plotted them as three separate clusters (Fig 2). The variations at the first and second axes were 28% and 13%, respectively. Analysis of similarity coefficient (ANOSIM r2 =0.624 at P < 0.001) was above 0.05 suggesting the data sets to be significantly different.

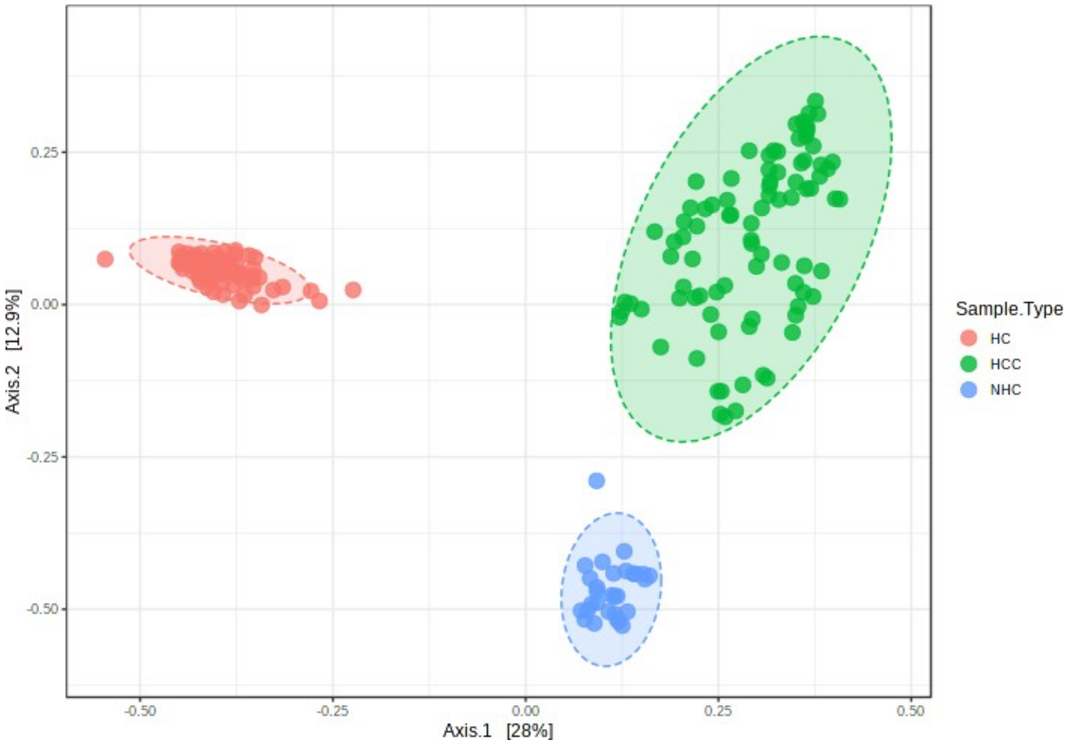

Fig S4. Beta diversity clustering based on PCoA on the Bray Curtis distances of bacterial genera in three different studies (HC-health care professionals from the present study presented as red cluster; HCC-health care professionals working in health care centre in Taiwan presented as green cluster; NHC-healthy individuals working in a livestock farm in Iowa presented as blue cluster)

<u>OTU filtering by MicrobiomeAnalyst</u>

The Marker data profile of Microbiome analyst was used. At first stage OTUs with ≥ 2 counts were removed filtering 1752 OTUs. Thereafter a minimum count filter of 4 was applied. In addition, the prevalence in samples was set at 20% and the Interquartile range set at 10%. This further removed 1580 low quality OTUs leaving behind 172 OTUs. All the subsequent analysis were performed on the set of 172 OTUs.

Table S3. Taxonomic profiling of the nasal bacterial community of health care professionals stationed in clinical research laboratory (a) Phlya (b) Class (c) Order (d) Family (e) Genus

Table S3a	Phyla	Abundance	Percentage
1	Firmicutes	2069594	13.09
2	Proteobacteria	4056536	25.65
3	Actinobacteria	3196194	20.21
4	Bacteroidetes	573721	3.63
5	Planctomycetes	1652	0.01
6	unclassified_ derived_from_Bacteria	5890939	37.25
7	unclassified_ derived_from_unclassified_sequences	25576	0.16

Table S3b	Class	Abundance	Percentage
1	Bacilli	1816038	11.48
2	Betaproteobacteria	21573	0.14
3	Gammaproteobacteria	2811401	17.78
4	Actinobacteria_ class	3196194	20.21
5	Alphaproteobacteria	1207027	7.63
6	Clostridia	253556	1.6
7	Flavobacteria	392077	2.48
8	Cytophagia	142453	0.9
9	Planctomycetacia	1652	0.01
10	Sphingobacteria	36831	0.23
11	unclassified_ derived_from_Bacteroidetes	2360	0.01
12	unclassified_ derived_from_Bacteria	5890939	37.25
13	Deltaproteobacteria	1450	0.01
14	unclassified_ derived_from_unclassified_sequences	25576	0.16
15	unclassified_ derived_from_Proteobacteria	15085	0.1

Table S3c	Order	Abundance	Percentage
1	Lactobacillales	188666	1.19
2	Burkholderiales	14039	0.09
3	Pseudomonadales	2352765	14.88
4	Ac@nomycetales	3202948	20.26
5	Rhizobiales	255055	1.61
6	Oceanospirillales	762	0
7	Chromadales	483	0
8	Clostridiales	253556	1.6
9	Bacillales	1629256	10.3
10	Caulobacterales	27478	0.17
11	Flavobacteriales	393873	2.49
12	Cytophagales	142453	0.9
13	Xanthomonadales	31929	0.2
14	Planctomycetales	1652	0.01
15	Enterobacteriales	230097	1.46
16	Sphingobacteriales	36831	0.23
17	Sphingomonadales	8608	0.05
18	unclassified_ derived_from_Bacteroidetes	2360	0.01
19	Rhodobacterales	1252	0.01
20	unclassified_ derived_from_Alphaproteobacteria	914634	5.78
21	unclassified_ derived_from_Bacteria	5887690	37.23
22	unclassified_ derived_from_Betaproteobacteria	7534	0.05
23	unclassified_ derived_from_Deltaproteobacteria	1450	0.01
24	unclassified_ derived_from_Gammaproteobacteria	186821	1.18
25	unclassified_ derived_from_unclassified_sequences	25576	0.16
26	unclassified_ derived_from_Proteobacteria	15085	0.1

Table S3d	Family	Abundance	Percentage
1	unclassified_ derived_from_Bacteria	5887690	37.23
2	Corynebacteriaceae	2997863	18.96
3	Moraxellaceae	2075436	13.12
4	Staphylococcaceae	1544058	9.76
5	unclassified_ derived_from_Alphaproteobacteria	914634	5.78
6	Flavobacteriaceae	393873	2.49
7	Pseudomonadaceae	277329	1.75
8	Clostridiales_Family_XI__Incertae_Sedis	253112	1.6
9	Enterobacteriaceae	230097	1.46
10	unclassified_ derived_from_Gammaproteobacteria	186821	1.18
11	Lactobacillaceae	171039	1.08
12	Cytophagaceae	142453	0.9
13	Rhizobiaceae	119602	0.76
14	Bradyrhizobiaceae	102954	0.65
15	Propionibacteriaceae	83369	0.53
16	Bacillaceae	73440	0.46
17	Micrococcaceae	68312	0.43
18	Sphingobacteriaceae	36225	0.23
19	Xanthomonadaceae	31929	0.2
20	Caulobacteraceae	27478	0.17
21	unclassified_ derived_from_unclassified_sequences	25576	0.16
22	Dietziaceae	23230	0.15
23	Methylobacteriaceae	19274	0.12
24	unclassified_ derived_from_Proteobacteria	15085	0.1
25	Aerococcaceae	14666	0.09
26	Phyllobacteriaceae	12204	0.08
27	Micromonosporaceae	10847	0.07
28	Sphingomonadaceae	8608	0.05
29	unclassified_ derived_from_Bacillales	8324	0.05
30	Comamonadaceae	7627	0.05
31	unclassified_ derived_from_Betaproteobacteria	7534	0.05
32	Nocardiaceae	5916	0.04
33	Microbacteriaceae	5041	0.03
34	Burkholderiaceae	4982	0.03
35	Brevibacteriaceae	2611	0.02
36	Planococcaceae	2526	0.02
37	unclassified_ derived_from_Bacteroidetes	2360	0.01
38	Streptococcaceae	2352	0.01
39	Planctomycetaceae	1652	0.01
40	Dermabacteraceae	1649	0.01
41	Intrasporangiaceae	1513	0.01
42	unclassified_ derived_from_Deltaproteobacteria	1450	0.01
43	Alcaligenaceae	1430	0.01
44	unclassified_ derived_from_Rhodobacterales	1252	0.01
45	AcJnomycetaceae	1237	0.01
46	unclassified_ derived_from_Rhizobiales	1021	0.01
47	Paenibacillaceae	908	0.01
48	Geodermatophilaceae	900	0.01
49	Enterococcaceae	609	0
50	unclassified_ derived_from_Sphingobacteriales	606	0
51	Halomonadaceae	479	0
52	unclassified_ derived_from_AcJnomycetales	460	0
53	Clostridiaceae	444	0
54	Alcanivoraceae	283	0
55	Ectothiorhodospiraceae	251	0
56	ChromaJaceae	*232*	*0*

Table S3e	Genus	Abundance	Percentage
1	*Abiotrophia*	1810	0.21
2	*Achromobacter*	138	0.02
3	*Acinetobacter*	1389	0.16
4	*AcJnobaculum*	15	0
5	*Aerococcus*	28	0
6	*Agrobacterium*	1969	0.23
7	*Alcanivorax*	16	0
8	*Alkalilimnicola*	15	0
9	*Aminobacter*	34	0
10	*Anaerococcus*	2071	0.24
11	*Arcanobacterium*	40	0
12	*Arthrobacter*	1060	0.12
13	*Bacillus*	6216	0.72
14	*Bosea*	1781	0.21
15	*Brachybacterium*	237	0.03
16	*Bradyrhizobium*	5404	0.63
17	*Brevibacterium*	148	0.02
18	*Brevundimonas*	2274	0.27
19	*Cellulophaga*	40	0
20	*Chryseobacterium*	560	0.07
21	*Clostridium*	46	0.01
22	*Corynebacterium*	124936	14.57
23	*Cytophaga*	13774	1.61
24	*Dietzia*	1955	0.23
25	*Elizabethkingia*	379	0.04
26	*Ensifer*	3683	0.43
27	*Enterococcus*	77	0.01
28	*Exiguobacterium*	607	0.07
29	*Finegoldia*	2444	0.29
30	unclassified_ deri	291	0.03
31	*Flavobacterium*	10489	1.22
32	*Gemella*	177	0.02
33	*Geobacillus*	98	0.01
34	*Geodermatophilu*	102	0.01
35	*Halomonas*	22	0
36	*Helcococcus*	93	0.01
37	*Hymenobacter*	55	0.01
38	*Ignatzschineria*	28	0
39	*Isosphaera*	169	0.02
40	*Kocuria*	831	0.1
41	*Lactobacillus*	7015	0.82
**Table S3e contd.**	Genus	Abundance	Percentage
42	*Lysinibacillus*	192	0.02
43	*Macrococcus*	166	0.02
44	*Mesorhizobium*	42	0
45	*Methylobacterium*	1021	0.12
46	*Microbacterium*	361	0.04
47	*Micrococcus*	3843	0.45
48	*Micromonospora*	932	0.11
49	*Moraxella*	88476	10.32
50	*Myroides*	7950	0.93
51	*Nesterenkonia*	47	0.01
52	*Paenibacillus*	74	0.01
53	*Pantoea*	1213	0.14
54	*Pedobacter*	699	0.08
55	*Pelomonas*	419	0.05
56	*Peptoniphilus*	6995	0.82
57	*Phyllobacterium*	947	0.11
58	*Planomicrobium*	108	0.01
59	*Propionibacterium*	4654	0.54
60	*Pseudomonas*	20630	2.41
61	*Rhodococcus*	507	0.06
62	*Rhodopseudomon*	64	0.01
63	*Riemerella*	296	0.03
64	*Rothia*	1229	0.14
65	*Shigella*	415	0.05
66	*Sinorhizobium*	96	0.01
67	*Sphingobacterium*	899	0.1
68	*Sphingobium*	249	0.03
69	*Staphylococcus*	88264	10.29
70	*Stenotrophomona*	2292	0.27
71	*Streptococcus*	226	0.03
72	*Tenacibaculum*	24	0
73	*Terrabacter*	64	0.01
74	*Terrimonas*	54	0.01
75	*Thiocapsa*	16	0
76	*Tropheryma*	18	0
77	*Ureibacillus*	72	0.01
78	*Xanthomonas*	160	0.02
79	*Xylella*	53	0.01
80	unclassified_ deri	228	0.03
81	Enterobacter	42	0
82	unclassified_ deri	5716	0.67
83	Klebsiella	1274	0.15
84	Ralstonia	206	0.02
85	unclassified_ deri	78	0.01
86	unclassified_ deri	105	0.01
87	unclassified_ deri	77279	9.01
88	unclassified_ deri	325038	37.91
89	unclassified_ deri	284	0.03
90	unclassified_ deri	114	0.01
91	unclassified_ deri	18813	2.19
92	unclassified_ deri	1100	0.13
93	unclassified_ deri	825	0.1

Core Microbiome analysis

Table S4 Core Microbiome Analysis

S. No.	Genera	Prevelance
1	***Corynebacterium***	0.89
2	***Staphylococcus***	0.75
3	***Pseudomonas***	0.56
4	***Moraxella***	0.44
5	***Myroides***	0.38
6	***Flavobacterium***	0.33
7	***Micrococcus***	0.29
8	***Propionibacterium***	0.27
9	***Peptoniphilus***	0.27
10	***Bradyrhizobium***	0.23
11	***Stenotrophomonas***	0.22
12	***Finegoldia***	0.21
13	***Brevundimonas***	0.19
14	***Cytophaga***	0.18
15	***Agrobacterium***	0.16
16	***Lactobacillus***	0.15
17	***Ensifer***	0.12
18	***Dietzia***	0.11
19	***Anaerococcus***	0.11
20	***Sphingobacterium***	0.10
21	***Bosea***	0.10
22	***Rhodococcus***	0.08
23	***Phyllobacterium***	0.07
24	***Pelomonas***	0.07
25	***Microbacterium***	0.07
26	***Bacillus***	0.07
27	***Shigella***	0.05
28	***Pedobacter***	0.05
29	***Kocuria***	0.05
30	***Chryseobacterium***	0.05
31	***Rothia***	0.04
32	***Pantoea***	0.04
33	***Methylobacterium***	0.04
34	***Klebsiella***	0.04
35	***Exiguobacterium***	0.04
36	***Acinetobacter***	0.04
37	***Abiotrophia***	0.04
38	***Sphingobium***	0.03
39	***Micromonospora***	0.03
40	***Streptococcus***	0.01
41	***Riemerella***	0.01
42	***Planomicrobium***	0.01
43	***Enterobacter***	0.01
44	***Elizabethkingia***	0.01
45	***Brachybacterium***	0.01
46	***Achromobacter***	0.01
47	***Xylella***	0.00
48	***Xanthomonas***	0.00
49	***Ureibacillus***	0.00
50	***Tropheryma***	0.00
51	***Thiocapsa***	0.00
52	***Terrimonas***	0.00
53	***Terrabacter***	0.00
54	***Tenacibaculum***	0.00
55	***Sinorhizobium***	0.00
56	***Rhodopseudomonas***	0.00
57	***Ralstonia***	0.00
58	***Paenibacillus***	0.00
59	***Nesterenkonia***	0.00
60	***Mesorhizobium***	0.00
61	***Macrococcus***	0.00
62	***Lysinibacillus***	0.00
63	***Isosphaera***	0.00
64	***Ignatzschineria***	0.00
65	***Hymenobacter***	0.00
66	***Helcococcus***	0.00
67	***Halomonas***	0.00
68	***Geodermatophilus***	0.00
69	***Geobacillus***	0.00
70	***Gemella***	0.00
71	***Enterococcus***	0.00
72	***Clostridium***	0.00
73	***Cellulophaga***	0.00
74	***Brevibacterium***	0.00
75	***Arthrobacter***	0.00
76	***Arcanobacterium***	0.00
77	***Aminobacter***	0.00
78	***Alkalilimnicola***	0.00
79	***Alcanivorax***	0.00
80	***Aerococcus***	0.00
81	***Actinobaculum***	0.00

Intra-personal Diversity

Table S5. Intra-personal variations among ten individuals sampled at three different time points

Genera	P1D	P1F	P1N	P10D	P10F	P10N	P2D	P2F	P2N	P3D	P3F	P3N	P4D	P4F	P4N
**Others**	0.4	0.0	0.0	0.3	0.0	0.0	0.0	0.0	0.0	0.2	0.3	0.1	0.1	0.0	0.1
Bacillus	0.0	0.0	0.0	0.1	0.0	0.0	0.0	0.0	0.0	0.0	0.0	0.0	0.0	0.0	0.0
Corynebacterium	0.0	0.1	0.1	0.0	0.0	0.2	0.0	0.2	0.0	0.0	0.1	0.0	0.0	0.1	0.4
Cytophaga	0.2	0.0	0.0	0.0	0.0	0.0	0.0	0.0	0.0	0.0	0.0	0.0	0.0	0.0	0.0
Flavobacterium	0.0	0.0	0.0	0.0	0.0	0.0	0.0	0.0	0.0	0.0	0.0	0.1	0.0	0.0	0.0
Moraxella	0.0	0.0	0.0	0.0	0.0	0.0	0.1	0.0	0.9	0.0	0.0	0.0	0.0	0.0	0.0
Myroides	0.0	0.0	0.0	0.0	0.0	0.0	0.0	0.0	0.0	0.0	0.0	0.0	0.0	0.0	0.0
Peptoniphilus	0.0	0.0	0.0	0.2	0.0	0.0	0.0	0.0	0.0	0.0	0.0	0.0	0.0	0.0	0.1
Pseudomonas	0.0	0.0	0.0	0.0	0.0	0.0	0.0	0.0	0.0	0.2	0.3	0.0	0.0	0.0	0.0
Staphylococcus	0.0	0.1	0.4	0.0	0.0	0.0	0.0	0.0	0.0	0.0	0.0	0.3	0.0	0.0	0.0
**unclassified_.derived_from_Alphaproteobacteria**	0.2	0.0	0.0	0.0	0.0	0.0	0.1	0.0	0.0	0.2	0.0	0.0	0.2	0.0	0.0
**unclassified_.derived_from_Bacteria.**	0.2	0.7	0.2	0.7	1.0	0.7	0.8	0.8	0.0	0.3	0.5	0.5	0.2	0.8	0.4
**unclassified_.derived_from_Gammaproteobacter**	0.3	0.0	0.0	0.0	0.0	0.0	0.0	0.0	0.0	0.3	0.0	0.0	0.4	0.0	0.0

Table S5 contd.

Genera	P5D	P5F	P5N	P6D	P6F	P6N	P7D	P7F	P7N	P8D	P8F	P8N	P9D	P9F	P9N
**Others**	0.1	0.1	0.0	0.0	0.0	0.0	0.2	0.0	0.0	0.6	0.3	0.1	0.3	0.3	0.1
Bacillus	0.0	0.1	0.0	0.0	0.0	0.0	0.0	0.0	0.0	0.0	0.3	0.0	0.0	0.0	0.0
Corynebacterium	0.0	0.0	0.4	0.2	0.5	0.6	0.0	0.0	0.1	0.0	0.0	0.1	0.0	0.1	0.0
Cytophaga	0.1	0.0	0.0	0.0	0.0	0.0	0.0	0.0	0.0	0.3	0.0	0.0	0.1	0.0	0.0
Flavobacterium	0.0	0.0	0.0	0.0	0.0	0.0	0.0	0.0	0.0	0.0	0.0	0.0	0.0	0.2	0.0
Moraxella	0.0	0.0	0.5	0.0	0.0	0.0	0.1	0.8	0.8	0.0	0.0	0.0	0.0	0.0	0.0
Myroides	0.0	0.0	0.0	0.0	0.0	0.0	0.0	0.0	0.0	0.0	0.0	0.0	0.0	0.1	0.0
Peptoniphilus	0.0	0.0	0.0	0.0	0.0	0.0	0.0	0.0	0.0	0.0	0.0	0.0	0.0	0.0	0.0
Pseudomonas	0.0	0.0	0.0	0.0	0.0	0.0	0.1	0.0	0.0	0.0	0.0	0.0	0.1	0.1	0.0
Staphylococcus	0.0	0.0	0.0	0.0	0.0	0.0	0.0	0.0	0.0	0.0	0.5	0.7	0.0	0.1	0.0
**unclassified_.derived_from_Alphaproteobacteria**	0.1	0.0	0.0	0.1	0.0	0.0	0.1	0.0	0.0	0.1	0.0	0.0	0.0	0.0	0.0
**unclassified_.derived_from_Bacteria.**	0.6	0.9	0.0	0.6	0.4	0.3	0.5	0.2	0.0	0.5	0.1	0.1	0.7	0.4	0.6
**unclassified_.derived_from_Gammaproteobacter**	0.2	0.0	0.0	0.0	0.0	0.0	0.0	0.0	0.0	0.0	0.0	0.0	0.0	0.0	0.0

P stands for Participant, the number represent Participant id and the Alphabet D stands for December, F is for February and N is for November

Hierarchical clustering

The hierarchical clustering feature of the microbiome analyst marker data profiling module was used to generate a heat map by applying the Ward algorithm on Euclidean distances. We noticed that the prevalence of all the genera differed with respect to season, age, gender, origin and season.

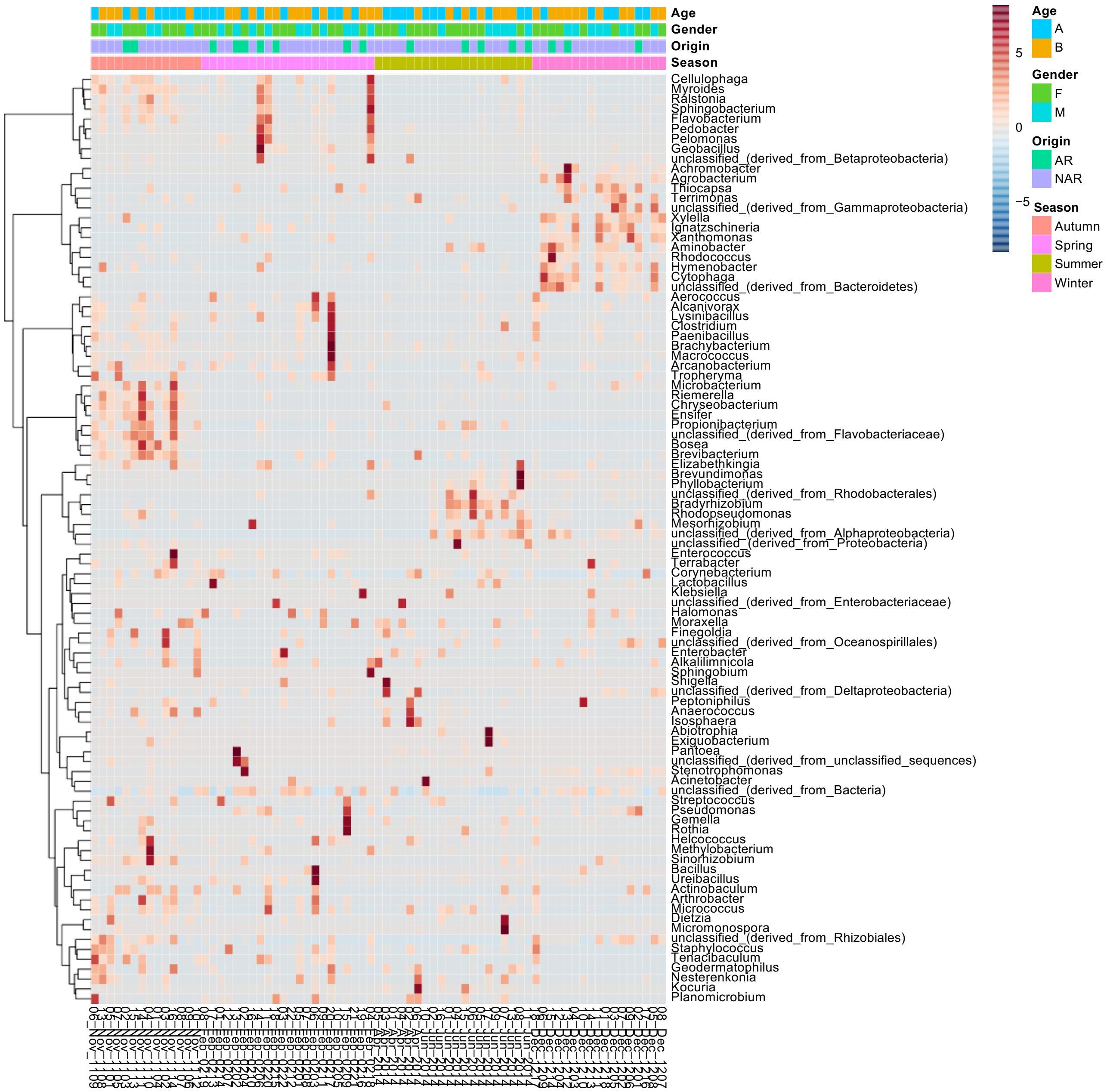

Fig S5. Hierarchical clustering based on seasonality, age, gender and ethnicity. Dark colored boxes represent the high prevalence of a particular genus in the respective sample.

Dendrogram Analysis

The dendrogram analysis corroborated the results of hierarchical clustering. All the samples formed a different branch indicating variations to be present within each sample.

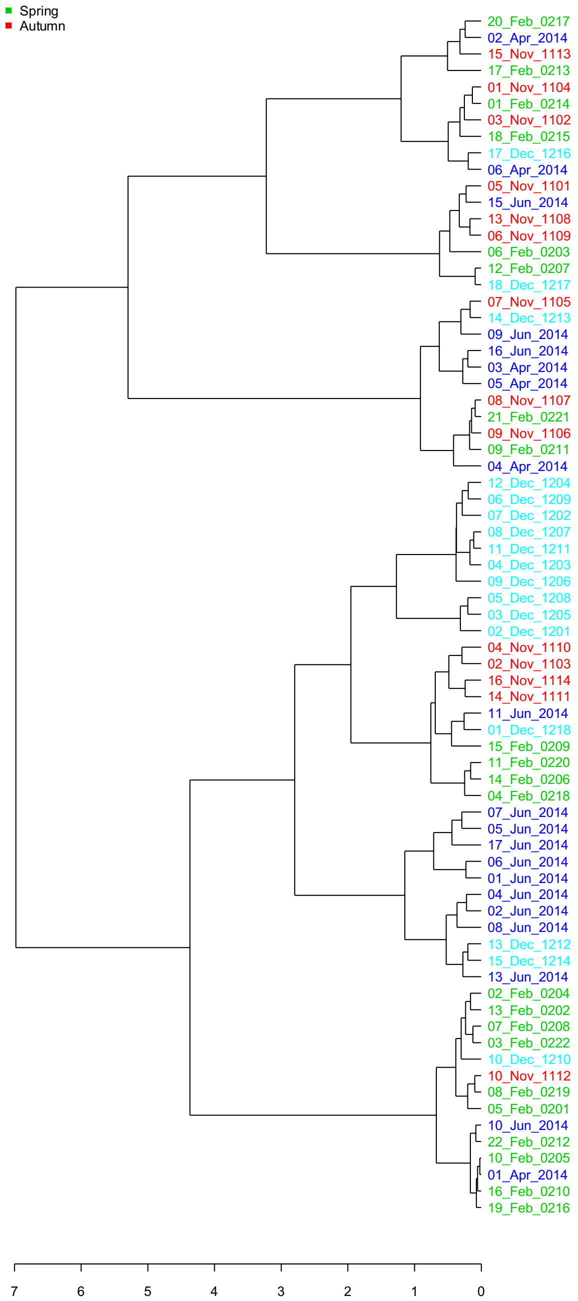

Fig S6. Dendrogram representing all the samples. Phylogenetic tree was constructed employing the Wardalgorithm on Bray Curtis Distances.

Beta Diversity Analysis/Community Structure Profiling

a

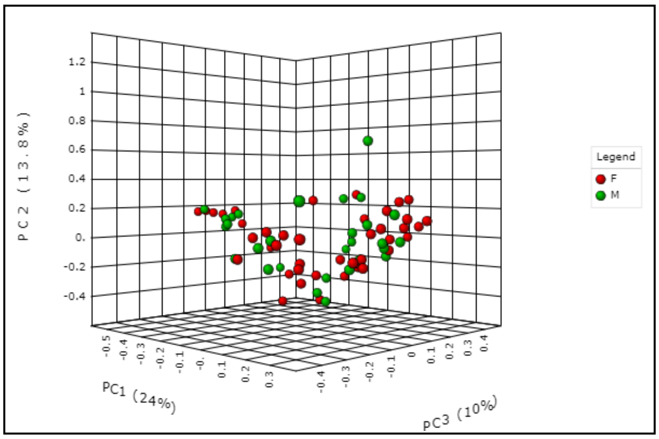

b

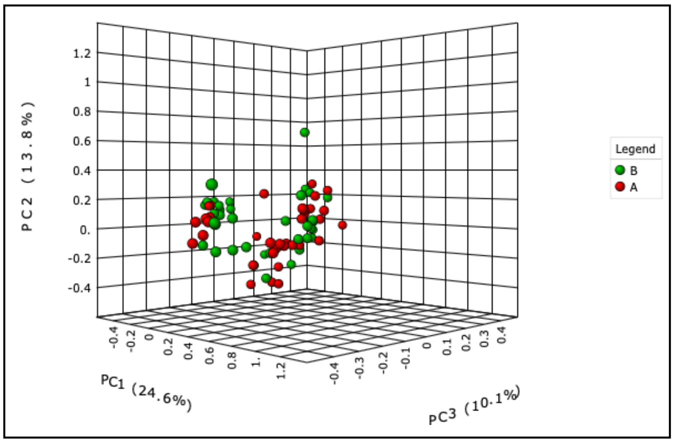

c

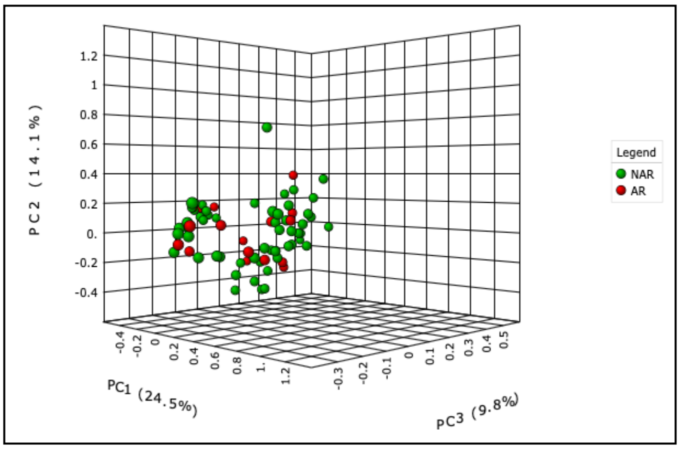

Fig S7. Beta diversity clustering based on PCoA on the Bray Curtis distances of bacterial communities with respect to (a) gender (b) age and (c) ethnicity. The samples were distributed into two genders namely male (M) and female (F). The age factor comprised of two groups 18- 30 years (B) and 30-60 years (A). Ethnicity included people of Arab (AR) and non-Arab (NAR) origin.
