# Supplementary material for: Composition of nasal bacterial community and its seasonal variation in health care workers stationed in a clinical research laboratory
Source: PLoS One. 2021 Nov 24;16(11):e0260314. doi: 10.1371/journal.pone.0260314 (PMC8612574; doi:10.1371/journal.pone.0260314)
Supplement: Attachment — Submitted filename: Response to Reviewers .docx [file pone.0260314.s002.docx]

PONE-D-21-17891
Composition of Nasal Bacterial Community and its Temporal Variation in Health Care Workers Stationed in a Clinical Research Laboratory
PLOS ONE

**Response to Academic Editors’ Comments**

Substantial revision of the English is required, with special attention to making sure terms are used accurately throughout the text. Both reviewers also raised concerns about the treatment of the control samples, which requires extensive clarification.

Thank you, Dr. Christopher Stanley, for your positive feedback and considering our manuscript for peer review.

Your suggestions on improvement of English language have helped us significantly to improve the legibility of the text. The English language has been edited through the Grammarly tool. It has also been read by a Native English Speaker in University of Manitoba.

We have keenly looked at the usage of terms such as relative abundance, prevalence and mean distribution and have made necessary changes wherever required for clarity of explanation. We have also provided a short definition of each term in the text.

While the experiments were conducted on health care professionals only and many of the analysis was completed after the project completion date, lack of a control group (non-healthcare professionals) was felt, we, therefore, used two publicly available datasets to compare our results with and use them as control.

All the changes in the manuscript have been marked in red.

We hope the manuscript is now fit for publication in PLoS ONE

**Response to Reviewers Comments**

**Reviewer #1 Prof. Amro Hanora**

We are thankful to Prof. Amro, for reviewing our manuscript and providing his valuable comments. This has helped us a lot to amend our manuscript. He has raised valid concerns and we have provided our response for each of his comments as given below.

1- The sentence in page 4 line 73 was a repeat of sentence in page 3 line 46

Response: Thanks for bringing this to our notice. We have replaced this section with new text (page 4; lines 77-81).

2- The sentence in page 4 line 87-88 (below 30 years and above 30 years of age), the age range need to verified with range.

Response: We agree with the reviewer’s suggestion and have now provided the range of age groups (page 4; line 90).

3- The design of the experiment in this work focused on the effect of indoor working environmental on nasal bacterial diversity, however, the authors does not explain or take in their account the effect both indoor environment at home and outdoor environment in their study. This can be achieved by using a control group from the same city outside the working environment, instead the authors use control group from different environment (20 healthy individuals (HC) working in a livestock farm in Iowa)

Response: The reviewer has raised a valid concern. Initially during the study only health care professionals were sampled. A control group was largely lacking. However, by the time the experiments on metagenomic analysis performed and complete analysis achieved the project was over. The study seemed to be incomplete without a control group and we felt that the results obtained cannot be referred to the microbiota of health care professionals. We therefore applied a strategy to compare our data with the data deposited in online repositories on nasal microbiota. We have compared our data with two studies, one is a group of health care professionals working in health care institutes of Taiwan (Chen et al., 2019) and the second is healthy individuals working in a livestock farm (OTUS from individuals not associated with animals) in Iowa (Kate et al., 2019). We downloaded the OTU files from these respective studies and merged it with our OTU table. We applied the centred log ratio normalisation on the entire dataset and performed the taxonomic profiling, linear discriminate analysis (LefSe) as well as the beta diversity clustering (PCoA). We discovered 1 genus to be significant differentially abundant among these studies. The beta diversity analysis plotted them as three separate cluster on the PCoA plot.

4- The authors enrolled 47 staff of the OMICS Research Unit/ Research Core Facility, Health Sciences Center, Kuwait University, Kuwait, or the staff from the departments in the same center. The indoor environment is different between different department, the author needs to explain why use individual diverse indoor environment as homogenous sample.

Response: The staff belonged to different department but during the study period they were using the research core facility to conduct their experiments/use the instruments and equipment available in the facility. The OMICSRU also provides working space to its users. The volunteers sampled were working in the same indoor environment as the staff of the facility during the present investigation and therefore the indoor working environment was considered homogenous. Text describing the OMICSRU location and usage has been added to the main manuscript (page 4; lines 93-96).

5- The authors need to explain why enrolled 10 individuals for intra personal diversity for three seasons (winter, spring and autumn) and does not include summer.

Response: Our aim was not to study the effect of seasonality among these individuals rather to see the differences in their microbiota at different time points. Three samples from the same individual at three different time points were considered as technically reasonable to study the intra-personal diversity and therefore, sampling was not done further.

6- The author needs to explain the working indoor environment for the 10-individual enrolled in intra personal diversity, as none of the individual posses the same bacterial profile.

Response: The 10 individuals worked on the first and second floor of the OMICSRU. Well, it was quite perplexing for us as well, that none of the individuals shared a common microbiome (page 18; line 440-443). We attributed this to diet, environment, host genetics, daily microbial exposure and personal habits (page 18; line 446-447). We have also added a table (Table 2) explaining the details of working locations of these individuals.

**Reviewer #2**

In the manuscript by Habibi et. al, the authors investigate the nasal microbiome of a cohort of workers in a clinical research laboratory. They collect samples across a single year, representing a snapshot of the microbiome at that time. They also longitudinally follow a small cohort of individuals and assess intra-person variability within those people.

It is clear that the nasal niche represents an important reservoir for bacterial colonization. Moreover, the bacteria that are found there can include opportunistic pathogens that can infect those that carry them, or be spread to other individuals. Thus, the topic is interesting and important. This being said, I found the presented details to be insufficient and the data analysis and presentation to be difficult to follow. I also think the authors need to be very careful about not over interpreting their results. Specific examples are provided below.

We express our gratitude to the reviewer for his positive feedback and finding the topic worthy of investigation and interesting. His thorough review and critical comments has significantly improved our manuscript. We have provided a point-by-point response to all his concerns in the text below:

Major comments:

1. The description of who was sampled and when is very confusing and needs to be clarified. The author’s clearly state that 47 individuals were included in the study. Of these, 10 individuals were sequentially sampled for 3 time points. Were the other 37 individuals only sampled once in one of the indicated seasons? If yes, that would suggest that the authors are looking at a total of 37+30=67 samples. However, this doesn’t match what’s presented in the paper or Table 1. Table 1 says that a total of 73 “individuals” were sampled in the 4 seasons. However, the other variables (age, gender, etc.) total to 75 “samples.” None of this makes sense and the authors need to clearly and precisely explain how many individuals were sampled, how many samples they got from each individual, etc. They also should make sure to not use the terms ‘individual’ and ‘sample’ interchangeably; these are different.

Response: We are apologetic of our ambiguous expression on the sampling details. We have modified the Table 1 completely for more clarity. We would like confirm that the total number of samples analyzed were 73. These 73 samples were collected from 47 individuals. The 10 individuals sampled thrice are a part of the 73 samples. The breakdown of samples collected in each season is provided in the modified Table 1. An additional table (Table 2) explaining their working location has been added to the manuscript.

2. In the methods, the authors mention several important controls that were included in the DNA isolation, 16S amplification and sequencing to account for any contamination. However, the results of what was obtained from those controls is not mentioned anywhere in the manuscript.

Response: The reviewer has raised a valid concern. Concerning the DNA isolation, the readings for DNA concentration from the plain swab as estimated by the Qubit HS dsDNA assay was too low, indicating absence of DNA. This statement has been provided in the manuscript (page-5 line 121). Regarding the 16 s amplification, the bioanalyzer trails of the positive control, negative control (nuclease free water), DNA Marker and sample library have been provided in the supplementary file Fig S1. Also, the Phred score of the libraries sequenced and analyzed through FASTQC have been mentioned in the supplementary table S1.

3. The authors need to include a detailed section on the sequencing results. How many reads were obtained? How many were left after filtering? How many reads were there per sample (range and median). Were all of the remaining reads used or was the data rarified in some way? None of these details are currently provided.

Response: We would like to bring to the reviewer’s attention that the details on sequencing reads were provided in the supplementary file Table 1. Data rarefaction was also performed and the rarefaction curves with Goods coverage was also provided in the supplementary data sheet Fig S1 (Main manuscript page 7; line168). The quality parameters of each sample can be assessed at the online MG-RAST repository. However, to give a quick glimpse on the quality parameters of the data to the readers we have added the total read counts and sequence counts to the main text in the manuscript. Page no. 8; lines 185-192.

4. The authors use the word “core microbiome” without ever really defining what that means. Are these found in 100% of samples? I don’t think so based on figure 2, which I believe suggests that some of their ‘core’ is in as little at 20% of samples? Why would this be considered ‘core’?

Response: We agree to the reviewer’s concern and we too found it difficult to set a cut-off sample size, as there was no rationale available for setting up the sample size. We followed the default settings of the software and obtained a heatmap with 12 genera showing prevalence above 20%. Very recently, the commentary published by Risely et al 2020 (new citation in the manuscript) has mentioned the sample size cut-off for host core microbiome as 30%. We have therefore, reworked this figure and modified the text accordingly (page 9; lines 211-219). The core microbiome analysis is based on genera recorded in more than 30% of the samples. The figure legend has also been modified accordingly.

5. I’m not sure the authors consistently use terms like ‘relative abundance’, ‘prevalence’, etc. Please make sure to clearly define what these terms mean within your study and then be consistent with their usage. Otherwise, it’s difficult to follow the results, descriptions, and figures.

Response: These are the terms obtained by default through the respective software. The relative abundance refers to the zero inflated microbial composition (page 8; line 199) collectively in all the samples, the prevalence means its occurrence in number of samples (page 9; line 215). The mean distribution is more or less similar to relative abundance. We have provided an explanation of these terms in the text.

6. For the seasonal variation, were all available samples included in this analysis? In other words, did this analysis include the 30 samples that came from the 10 individuals that were sampled in the 3 sequential months? What’s included should be clearly stated.

Response: The seasonal variation was studied for all the samples (including the 30 samples for intra-personal diversity) sampled in the 3 sequential months. The table describing the sampling details has been modified and specifies the exact number of samples for each experimental parameter.

7. The sampled individuals were listed as healthy, but were they healthy for the entire year of the study? Did any of them take antibiotics at any point within the year? If yes, this could certainly account for variability. Were there any exclusion criteria that were used to select participants?

Response: The reviewer has raised a valid concern. The word healthy here signifies that the sampled individuals did not suffer from any upper respiratory tract infections and were not on any antibiotics pertaining to it during the entire study period. In fact, higher number of volunteers (than mentioned in the manuscript) were registered during the study period and if anyone reported the intake of antibiotics, were not sampled further and excluded from the study. A statement relevant to the intake of antibiotics has been added to the manuscript text. Page no 5 lines 105-106.

8. The authors repeatedly state that their data show that temperature/season are the driving forces resulting in nasal microbiome changes. These statements need to be softened. Realistically, these individuals spend the majority of their time outside of the work environment and may be exposed to any number of external exposures or environments that may affect the microbiome. Thus, the authors should not overstate their results. Temperture/season seems to be important in the very small number of variables that you assessed.

Response: We totally agree with the reviewer’s suggestion. We had no intentions to overstate the results. We have revisited the result and discussions section and rephrased the text to remove the impression of overstatement.

9. I think that all of the legends should be expanded to very clearly explain what is shown/found in the figures and tables. Make sure your terminology is consistent and accurate.

Response: Modification have been done as suggested

Specific comments:
1. Line 22: Why are these individuals ‘potentially healthy’? This is an odd phrase.

Response: We have removed the word “potentially” (page 2; line 25)

2. Line 27: Were these different ‘in all 4 seasons’ or actually ‘different across the 4 seasons’. For the first to be true, you would have to observe significant differences in each season when compared to each of the other seasons. I’m not sure that’s what the data show.

Response: We mean to say different across the 4 seasons. We have rephrased this sentence on page 2, line 30

3. Line 44: change to read “…tract and are involved in the….”

Response: Changed as suggested (page 3; line 48)

4. Lines 73-79 are virtually identical to what is presented in the preceding paragraphs and should be deleted or edited.

Response: We somehow overlooked the redundancy here. We have replaced the text with new information (page 3; lines 77-81).

5. Line 88: I found the statement of age to be very confusing. Upon looking at the table, I realized the authors were saying that they grouped the individuals into two categories (<30 years or >30 years). They really should provide the age range of the sampled individuals and then state that they broke them into groups to assess the potential importance of age on the nasal microbiome.

Response: We agree to the reviewer’s response. We have rephrased our statement and specified the range of age groups categorized in two groups A and B (page 4; line 90).

6. Lines 99-101 and elsewhere: The authors should not call these ‘controls’. These are not controls, but are examples of different data sets that you compared your data to as a way to determine how comparable the results were.

Response: We have rephrased this statement and removed the word control from the text (Page 5; line 108).

7. Line 111: change to read “…processing for subsequent 16S amplification.”

Response: Changed as suggested (page 5; line 119)

8. Line 114: the ‘S’ should be capitalized in 16S.

Response: Changed as suggested (page 6; line 123)

9. Line 116-117: Do these primers have names? Please provide names in addition to sequences.

Response: These primers were designed as per the 16S amplification application note of Illumina. However, the primers recommended by Illumina are based on the most promising primer pair S-D-Bact-0341-b-S-17/S-D-Bact-0785-a-A-21 identified by Klindworth et al., 2013. This information has been added to the text (page 6; lines 124-129).

10. Line 155: the word ‘filtered’ implies that the 2363 OTUs were discarded from the analysis. I don’t think that is what the authors mean. I think they are saying that that many OTUs remained after all of the filtering steps.

Response: Thanks for bringing this to our notice. We have replaced the word ‘filtered’ by ‘picked'. (Page 7; line 166)

11. Line 181: Do the authors really classify at the ‘species’ level? This is difficult to do with such short reads. If they were able to classify some sequences (OTUs) to the species level, I saw no real discussion of various species that they identified.

Response: We did the profiling upto the species level, however as the 16Samplicon is not an appropriate technique to classify the sequences upto the taxon therefore, we did not mention any of these results. To avoid any confusion, we are removing this statement from the text (page 8; line 200).

12. Lines 297-298 as compared to Lines 314-315. Aren’t these two results/statements conflicting?

Response: We had performed the hierarchical clustering and dendrogram analysis which usually returns a heat map and a branched tree. A quick scan at these two figures provides first-hand information on variations in the samples if any. We have rephrased the statement. (Page 13; lines 320-322)

13. Lines 360, 397, and elsewhere: terms like ‘disease condition’ are vague. State the disease that you are referring to.

Response: Names of the diseases have been provided. (Page 15, line 382; page 17, line 423)

14. Line 443: Change to read “…possesses a stable personalized microbiome….”

Response: Changed as suggested (page 19’ line 471)

15. Line 460: ‘tree by’.

Response: The legend has been rephrased

16. Figure 11 panels B, C and D could be moved to the supplement since panel A is the only one that showed an interesting result.

Response: We respect the reviewer’s suggestion and have modified the fig 11. We have moved the panels B, C and D to the supplementary file section. We have added the 2D PCoA plot for better explanation of the clusters.

17. I think the authors should be careful with the use of the terms ‘longitudinal and temporal.’ I would agree that the 10 individuals that were sampled in sequential months were followed longitudinally. ‘Temporal’ can also imply the same thing as longitudinal, meaning you followed something across time. Maybe “seasonal” would be a better word since you don’t appear to sample most of the individuals more than once? This could be altered in the title.

Response: We agree with the reviewer’s recommendation and have modified the title as suggested.
