# Supplementary material for: Composition of nasal bacterial community and its seasonal variation in health care workers stationed in a clinical research laboratory
Source: PLoS One. 2021 Nov 24;16(11):e0260314. doi: 10.1371/journal.pone.0260314 (PMC8612574; doi:10.1371/journal.pone.0260314)
Supplement: Attachment — Submitted filename: Response to reviewers copy.docx [file pone.0260314.s003.docx]

**Response to reviewers**

Reviewer #2: The manuscript by Habibi et al., is a revised version of a manuscript that I previously reviewed. The authors have made attempts to address the prior comments, and the work is improved. However, there are still issues with the writing and some of the presented information.

Thank you for reading our manuscript again. We are grateful for your constructive comments. This, has once again improved our manuscript.

1. One of my prior concerns was with the authors failure to adequately explain where all of the samples came from. To address this, they have added Table 1. This table shows the distribution of the number of samples that fell into the various epidemiologic groupings. The label over the right hand side of the data states “No of individuals sampled” and shows that these values all add up to 73 across the various breakdowns. However, the authors state in the paper that they sampled from “47” individuals. They seem to be confusing ‘samples’ and ‘individuals.’ Thus, I still have no clue how they arrived at 73 samples from 47 individuals. 10 individuals were sampled 3 times each, which would be 30 samples. If the remaining 37 individuals were sampled only once each, that would be 37 samples. 30+37=67 and not 73. Something is clearly not explained clearly or accurately. This needs to be clarified.

Response: We would like to inform the reviewer that 73 samples came from 47 healthy volunteers only. From the remaining 37 individuals few were sampled more than once, making the total number of samples to 73. This information has been added to the methodology section. Lines 91-92. Also, the heading in column 4 in Table 1 has been changed from no of sample to total no. of individuals sampled in each season.

2. I think point #1 indicates that the work is still in need of significant language editing. The only language changes that were made appear to be in response to specific comments. Judicious editing would help with clarity and the presentation of the work; you want it to be as interpretable by the audience as possible. See a few of the specific comments below.

Response: We accepted the reviewers suggestion. The manuscript has now been read by a native English speaker (Ms. Christiana Cholakis, from University of Manitoba) and is hopefully free from linguistic errors.

3. The sections added on lines 185-192 are poorly written and difficult to follow.

Response: This section has been rewritten.

4. I still don’t think the authors have clearly defined in the paper what they consider to be a ‘core’ microbiome. The easiest way to address this is that the first time this phrase is use, the authors should state something along the following: ‘we defined the core microbiome as genera present in 30% of the individual samples’. If ‘genera’ is not correct, add the correct word. They can also include the reference they mention in their response to my prior comment.

Response: We have added this phrase in the methodology section with the relevant citation (Line 208-209). We have also done some changes in the text to be clearer on this aspect. For eg. Lines 106, 223, 603, 605 have been re-phrased.

5. Lines 111. Was there a set procedure or is there a published procedure for how the swab was collected? Was it inserted into a particular nostril first, twirled a certain number of times, etc.? Provide information or a reference.

Response: A reference to the swab collection procedure has been added (Line 111)

6. Line 194-195. This may not be true. You have no way of knowing that this difference has anything to do with these individual’s occupations. It may simply be due to geographic differences of the sampled populations, etc.

Response: This sentence has been reframed and the reason of spatial variations has also been added. Line 194-195.

Specifics:
1. The sentence beginning “Competition….” On line 23 seems incomplete. I’m not sure what you are trying to say.

Response: We have modified this sentence.

2. It’s not clear how someone can “indirectly” work in a clinical research lab (line 25).

Response: We have removed the words directly and indirectly.

3. Line 38. Captalize “S” in 16S

Response: The change has been done

4. Line 42. Add a comma after “ecosystem”

Response: The requested change has been done

5. Line 53. “the key medium” should likely be “a key medium”

Response: The has been replaced by ‘a’

6. Line 54. “structure” should be “structures”

Response: The change has been done.

7. Line 60. The effect of what? The meaning is unclear.

Response: This sentence has been removed from the paragraph

8. Line 64. “disperse” should be “disperses”

Response: The amendment has been done

9. Line 166. “picked” doesn’t seem like the best word. Perhaps “revealed a total of 2363 OTUs withing the dataset.”

Response: We have replaced picked with the suggested phrase

10. These are only a few examples of the language issues that I’m talking about in the the major point #2 above. Virtually every paragraph has many examples like this that need to be fixed.

Response: We are once again apologetic of our ambigous expressions. The manuscript has been read by an English native speaker and is hopefully free from linguistic errors.
