# Supplementary material for: Composition of nasal bacterial community and its seasonal variation in health care workers stationed in a clinical research laboratory
Source: PLoS One. 2021 Nov 24;16(11):e0260314. doi: 10.1371/journal.pone.0260314 (PMC8612574; doi:10.1371/journal.pone.0260314)
Supplement: Attachment — Submitted filename: Response to Reviewers.docx [file pone.0260314.s004.docx]

**Response to Reviewers**

Reviewer #2: This is the 3rd version of this manuscript that I have reviewed. This version is much improved and I appreciate the authors efforts to address all of my prior concerns.

Response: We are very much thankful to the anonymous reviewer for his encouraging feebback.

Reviewer #3: In this article, the authors characterized the structure and dynamics of nasal microbiome from healthy individuals in a clinical research laboratory in Kuwait. The authors also investigated factors driving the variation in nasal microbiome in this special population.

In general, it is easy to capture the main points of this paper. However, the authors need to clarify the samples they used or reconsider some statistical methods before drawing some of their conclusions.

Response: We express our gratitude to the reviewer (Dr. Nan Shen) for his positive feedback. We have provided a response to his valid concerns in the following text.

Major issues:

1.Page 5 line 107-110, “The dataset from 88 professionals working in health care centres (HCC) in Taiwan and 20 healthy individuals (HC) working in a livestock farm in Iowa were used to compare our dataset with healthy individuals working in non-healthcare settings”. Page 8 line 195-196, “These variations were presumed due to the differences between the nasal bacterial communities of health care and non-healthcare workers.” 88 of them were working in a health center in Taiwan. I suppose they are health care workers. Why take them as part of the non-healthcare controls? Both ethnic or genetic and climate (Taiwan is tropical and Kuwait is desert climate) will be the main drivers on their nasal microbiome, especially as the authors have found the seasonal change in their samples. The other 22 samples were from a farm in the US. Exposure to the livestocks and outdoor jobs will make a great difference to their nasal microbiome. These two groups were not good controls if the authors want to demonstrate the difference between healthcare workers v.s. non-healthcare workers.

Response: We are in agreement with the reviewer on this aspect. As the study lacked control samples from Kuwait (Where the whole project was executed) we applied the strategy to use publicly available datasets for comparison. The studies on nasal microbiome of healthy subjects were limited, however we were able to get these two datasets. As pointed out by the reviewer and also our own understanding, Taiwan and Kuwait are two distantly apart countries and differences in the nasal microbiome of health care workers owing to the geographical differences are obvious, we are removing the word control for this dataset.

Regarding the other dataset *i.e.*  healthy workers (NHC) in a farm. We see the farm as a different setting than that of a health care centre. Also, these individuals were not in contact with animals on the farm and were mostly working indoors. The differences in community composition can therefore be attributed to the difference in workplace and therefore we believe it can be used as a control. We cannot ignore the environmental factors as well and this explanation has also been given in the text. (page 5 Lines 108-112; page 8 lines 195-204)

2.Page 10-12 Session “seasonal variation”. Which samples were the analysis done with? If the recruited subjects were sampled for different times, the final results will be driven by the individuals sampled more times, making the conclusions unreliable. The best practice is to conduct paired analysis for differential comparison and track the change within the subject. For the same reason, correlation between bacteria abundance across samples is not appropriate for identifying co-occurring bacteria. Consider similarity in patterns of longitudinal change instead.

Response: The analysis was performed on all the samples. Although, the individuals were sampled more than once, however were not repeated in the same season. In addition to this almost 50% of subjects sampled in a particular season were unique and therefore, we believe that the variations are due to seasonality. We would also like to bring to the reviewer’s attention that pairwise comparisons on alpha diversities were already done through ANOVA an analogue of paired t-test. However, an additional comparison between summer vs winter; summer vs spring; summer vs autumn; winter vs spring; winter vs autumn and spring vs autumn has been performed. The differential abundance analysis on non-parametric Wilcoxson rank sum test at a confidence interval of p<0.05 was used for this. This compared the median abundances within a particular season. The results have been added in the manuscript (Page 11 Lines 268-285 and new fig 6).

3. A comparison between inter-subject dissimilarity and inter-factor dissimilarity will help the audience to understand how strong the influence of this factor is for all the analysis to investigate factors driving variation in nasal microbiome. Also, the authors have shown the instability of nasal microbiome within subjects. But, I am not sure how unstable it is. Is it still possible to tell if two samples were the same subject?

Response: Thank you for bringing this to our notice. We have done the pairwise comparisons employing a student’s t-test on two variables that are inter-subject dissimilarity and inter-factor dissimilarity. All the results were highly significant p-value < 0.05. The variance table has been provided in the manuscript. The nasal microbiome differed significantly as proven by the t-test. (page 13 lines 328-331; table 4)

Taking apart the prevalent genera, a good number of other genera were found in each subject when sampled at a different time point. Therefore, we assume that the microbiome might not be same for the same subject if sampled at a different time point.

Minor issues:

1. Page 2 line 28, “The taxonomic profiling and core microbiome analysis predicted three predominant genera as Corynebacterium (15.0%), Staphylococcus (10.3%) and, Moraxella (10.0%).” Unless the authors have tested this conclusion on unseen data, “predict” is not a suitable verb here.

Response: This word has been replaced by identified. (page 2Line 28)

2. Page 2 line 45, “skin, blood, urine and, any other crevices or orifices.” Bacteria in urine come from the bladder or urinary tract, like fecal microbiome come from the gut.

Response: blood and urine have been removed from this sentence (Page 2 line 44-45)

3. Page 8 line 177, as multiple samples collected from the same subjects, paired statistical analysis methods will be more appropriate.

Response: Please see the response 2 and 3 under major issues.

4. Page 8 line 179-180, Bray-curtis is not a good choice for compositional data dissimilarity. Besides, Bray-curtis should be between 0 to 1 and it's ratio between arithmetic sums. It cannot be used in CLR transformed data. CLR is in log scale, it's geometric and CLR data will have negative values.

Response: We have opted for the Bray Curtis dissimilarity based on several studies employing this method. We still prefer to use it. We have now used the TSS method for data normalization. The values and the figures have been replaced accordingly. (Page 6; line 183; page 14 line 353-354, fig 13)

5. Page 8 line 191, “Data was rarified (Fig S3) and sequences < 5 bases and quality score < 20, were filtered out.” Did the authors rarified before the quality filter?

Response: Data was first processed for quality check and then rarified. We have done this change in the text. (page 8, line 194)

6. Page 9 line 206-208, why is the RA of Actinomycetales higher than the RA of Actinobacteria?

Response: This was a typo error and has been resolved. Page 9 line 215

7. Page 13 line 304-305, “From these observations, we concluded that an individuals’ nasal microbiome is largely defined by the prevailing season in addition to personal habits and daily routine.” Is there any evidence in this study to support the impact of “personal habits and daily routine” on nasal microbiome?

Response: we have replaced the word concluded with assumed. Page 14; line 332

8. Page 13 line 318-329. In the alpha diversity analysis, the autumn microbiome stood out among the four seasons. But in the beta diversity, it’s quite the opposite. What can be the reason?

Response: Alpha diversity measures within the group diversity, whereas beta diversity is measuring among the group diversity. In autumn season the species richness, evenness and abundances might be high whereas these population might be overlapping with other seasons. This is the only reason we think are responsible for the differences in alpha and beta diversities of autumn season.
